# Whole-Exome Sequencing Analysis of Inflammatory Bowel Disease-Associated Serrated Dysplasia

**DOI:** 10.3390/ijms26125704

**Published:** 2025-06-13

**Authors:** Zsófia Balajthy, Szintia Almási, Tamás Lantos, Levente Kuthi, Georgios Deftereos, Won-Tak Choi, Anita Sejben

**Affiliations:** 1 Department of Pathology, Albert Szent-Györgyi Medical School, University of Szeged, 6725 Szeged, Hungary; 2 Department of Medical Physics and Informatics, Albert Szent-Györgyi Medical School, University of Szeged, 6720 Szeged, Hungary; 3 Department of Surgical and Molecular Pathology, Tumor Pathology Center, National Institute of Oncology, 1122 Budapest, Hungary; 4 Department of Pathology and Experimental Cancer Research, Semmelweis University, 1085 Budapest, Hungary; 5 HUN-REN-ONKOL-TTK-HCEMM Oncogenomics Research Group, National Institute of Oncology, 1122 Budapest, Hungary; 6 Department of Pathology, University of California at San Francisco, San Francisco, CA 94143, USA

**Keywords:** dysplasia, inflammatory bowel disease, nonconventional, serrated dysplasia, sessile serrated lesion, traditional serrated adenoma

## Abstract

The clinicopathologic and molecular features of serrated lesions with dysplasia in inflammatory bowel disease (IBD) remain poorly understood. We examined a total of 2396 patients treated for IBD at the University of Szeged between 2011 and 2023. Among them, 177 (7%) patients were diagnosed with colorectal neoplasia, of which only 11 (6%) had serrated dysplasia (n = 13). Of the 13 lesions, 5 (38%) showed features of sessile serrated lesion (SSL)-like dysplasia; 1 (8%) exhibited characteristics of traditional serrated adenoma (TSA)-like dysplasia; 6 (46%) were classified as serrated dysplasia, not otherwise specified (NOS); and 1 (8%) displayed mixed features of SSL-like and TSA-like dysplasias. At the time of the serrated dysplasia diagnosis, the mean age of the patients was 56 years. Ten (91%) patients had ulcerative colitis, and one (9%) had Crohn’s disease. Pancolitis was observed in seven (64%) patients. The mean duration of IBD at the time of the serrated dysplasia diagnosis was 26 years. Most lesions (n = 9; 69%) were found in the left colon, including SSL-like dysplasia (3/5; 60%) and serrated dysplasia NOS (5/6, 83%). Eleven (85%) lesions had a polypoid endoscopic appearance. The mean size of the serrated dysplasia was 0.8 cm. Most lesions (n = 8; 62%) showed low-grade dysplasia. Serrated dysplasia was often associated with conventional (n = 3; 27%) or nonconventional dysplasia (n = 3; 27%). During the follow-up, 5 (45%) of the 11 patients developed colorectal cancer, including 3 patients with serrated dysplasia NOS, 1 with SSL-like dysplasia, and 1 with TSA-like dysplasia. Whole-exome sequencing revealed that the SSL-like dysplasia harbored mutations in *BRAF* (p.V600E), *MLH1, KRAS, PTEN, POLE, KMT2C,* and/or *EXT1*, whereas the serrated dysplasia NOS showed mutations in *TP53, POLG, BRAF* (p.G469A), *KMT2C*, and/or *EXT1*. One patient with both SSL-like dysplasia and mixed SSL-like/TSA-like dysplasia carried a pathogenic *MUTYH* (p.R217H) mutation, along with mutations in *MADD*. Serrated dysplasia was rare in IBD, with a prevalence rate of 6%. The SSL-like dysplasia exhibited distinct clinicopathologic and molecular characteristics compared with its sporadic counterpart. Similarly, serrated dysplasia NOS displayed unique molecular features compared with SSL-like dysplasia and could carry a higher risk of malignancy.

## 1. Introduction

There are 3 distinct serrated subtypes with dysplasia (hereafter referred to as serrated dysplasia) in inflammatory bowel disease (IBD), including sessile serrated lesion (SSL)-like dysplasia; traditional serrated adenoma (TSA)-like dysplasia; and serrated dysplasia, not otherwise specified (NOS) [1,2,3,4,5,6,7,8,9,10,11,12,13,14]. SSL-like dysplasia is defined by the presence of dilated L-shaped or inverted T-shaped crypts at the interface with the muscularis mucosae, with features of dysplasia. TSA-like dysplasia often shows a tubulovillous or villous growth pattern, characterized by low-grade columnar cells with eosinophilic cytoplasm, slit-like serrations, and ectopic crypts. Serrated dysplasia, NOS demonstrates a complex serrated morphology with dysplasia that does not meet the diagnostic criteria of SSL-like dysplasia or TSA-like dysplasia.

Although the clinicopathologic and molecular features of serrated dysplasia, NOS are largely unknown, earlier studies demonstrated that SSL-like dysplasia and TSA-like dysplasia may share similar clinicopathologic and molecular features with their sporadic counterparts [2,5,6,7,8]. For instance, SSL-like dysplasia is typically found in the right colon, whereas TSA-like dysplasia is more common in the left colon [2,5,6]. Also, low-grade serrated dysplasia, which often resembles sporadic TSA, frequently demonstrates *KRAS* mutations (45% vs. 18% for *BRAF* mutations) [5]. However, due to their low incidence, with each serrated dysplastic subtype accounting for only 1% of all dysplastic lesions in IBD, the characterization of these dysplastic lesions has been limited. Therefore, this study aimed to further characterize these lesions and investigate their molecular features via whole-exome sequencing (WES).

## 2. Results

**Clinicopathologic features of serrated dysplasia—Table 1** summarizes the clinicopathologic features of the 11 IBD patients who developed 13 serrated dysplastic lesions. The mean age at the time of the serrated dysplasia diagnosis was 56 years (range: 35–71). Serrated dysplasia occurred predominantly in men (n = 8; 73%). Ten (91%) patients were diagnosed with UC, and 1 (9%) patient had CD. Pancolitis was observed in 7 (64%) patients, whereas the remaining 4 (36%) patients had left-sided colitis. The mean duration of IBD at the time of serrated dysplasia diagnosis was 26 years (range: 5–59). No patient had a concurrent history of PSC.

**Figure 1:**
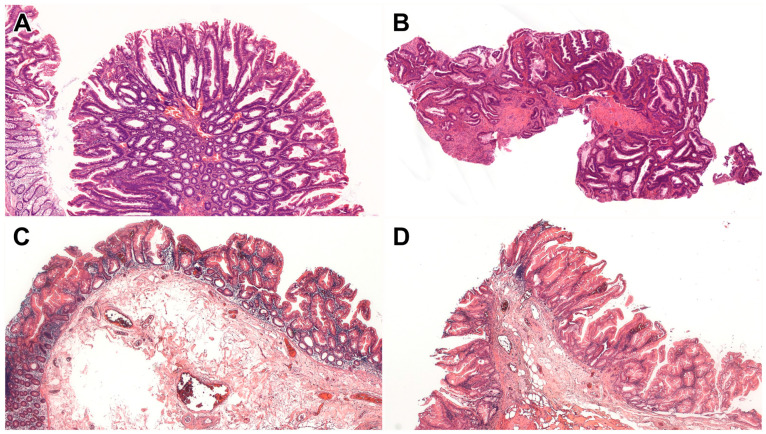
Serrated dysplastic subtypes. (**A**) SSL-like dysplasia shows dilated crypts at the interface with the muscularis mucosae, accompanied by a dysplastic epithelium (HE, 5×). (**B**) Serrated dysplasia NOS demonstrates a complex serrated architecture with dysplasia. There is no definite evidence of SSL-like dysplasia or TSA-like dysplasia (HE, 5×). (**C**,**D**) In mixed SSL-like/TSA-like dysplasia, one area shows SSL-like dysplasia characterized by dilated L-shaped or inverted T-shaped crypts at the interface with the muscularis mucosae (**C**) (HE, 5×), while another area shows TSA-like dysplasia featuring a tubulovillous growth pattern, eosinophilic cytoplasm, and slit-like serrations (**D**) (HE, 5×). Abbreviations: HE, Hematoxyilin and eosin; NOS, not otherwise specified; SSL, sessile serrated lesion; TSA, traditional serrated adenoma.

Of the 13 serrated dysplastic lesions, 5 (38%) exhibited characteristics of SSL-like dysplasia, 1 (8%) showed features of TSA-like dysplasia, 6 (46%) were classified as serrated dysplasia, NOS, and 1 (8%) displayed mixed features of SSL-like and TSA-like dysplasias (Table 1 and Table 2; Figure 1). Most lesions (n = 9; 69%) were found in the left colon, including SSL-like dysplasia (3/5; 60%) and serrated dysplasia, NOS (5/6, 83%). Eleven (85%) lesions had a polypoid endoscopic appearance, while the remaining 2 lesions, including 1 serrated dysplasia, NOS and 1 mixed SSL-like/TSA-like dysplasia, had a flat endoscopic appearance. The mean size of the lesions was less than 1 cm, except for the serrated dysplasia, NOS (mean: 1 cm, range: 0.3–2.5). The SSL-like dysplasia predominantly showed low-grade dysplasia (4/5; 80%), whereas half of the serrated dysplasia NOS cases (3/6; 50%) displayed high-grade dysplasia (HGD). Among the 5 patients with either SSL-like dysplasia or serrated dysplasia, NOS, 1 (20%) also presented with conventional dysplasia. However, nonconventional dysplasia was more frequently associated with the serrated dysplasia, NOS (3/5; 60%) than with the SSL-like dysplasia (1/5; 20%). During the follow-up, 5 (45%) of the 11 patients developed CRC, including 3 patients with the serrated dysplasia, NOS, 1 with the SSL-like dysplasia, and 1 with the TSA-like dysplasia. The median progression-free survival was 22 months (range: 1–64) in the SSL-like dysplasia group and 15.8 months (range: 1–51) in the serrated dysplasia, NOS group, and proved to be 1 month in the patient diagnosed with TSA-like dysplasia and 36 months in the mixed SSL-like/TSA-like dysplasia patient. In the case of the median overall survival, 34.6 months (range: 1–64) and 41 months (19–73) were established in the SSL-like dysplasia and serrated dysplasia NOS groups, respectively, while 17 and 51 months were recorded for the TSA-like dysplasia and the mixed SSL-like/TSA-like dysplasia patients.

**Figure 2:**
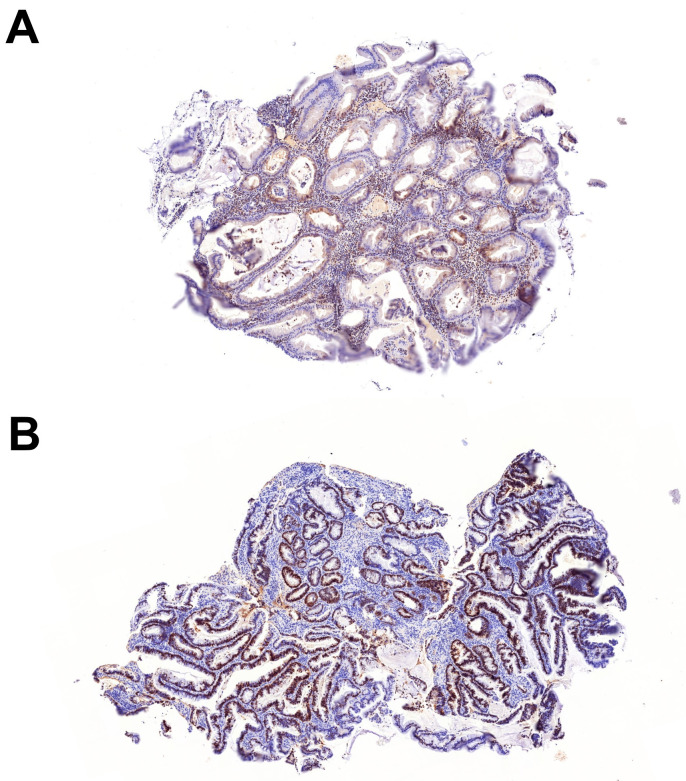
Immunohistochemical analysis of SSL-like dysplasia and serrated dysplasia NOS. (**A**) MLH1 immunohistochemistry showed a loss of staining in SSL-like dysplasia (5×). (**B**) p53 immunohistochemistry demonstrated overexpression in serrated dysplasia NOS (5×).

**Molecular features of serrated dysplasia**—WES was performed on 8 serrated dysplastic lesions from 7 patients (Table 3). For the remaining 5 lesions, either paraffin blocks were unavailable or the samples were unsuitable for WES. The SSL-like dysplasia in patient #5 harbored a likely pathogenic variant in *MLH1*, along with a high number of pathogenic and likely pathogenic variants, most of which were small frameshift insertions or deletions, which is suggestive of a microsatellite instability (MSI). Among these, a *KRAS* p.G12C mutation and 2 mutations in *PTEN* were identified. Consistent with the MLH1 mutation in patient #5, the immunohistochemistry for MLH1 and PMS2 showed a loss of staining, confirming MSI (Figure 2A). Also, the SSL-like dysplasia in patient #3 showed an inactivating variant in *POLE*; however, this lesion did not exhibit a high number of mutations, suggesting that this variant is likely a passenger mutation rather than a driver mutation. Furthermore, the SSL-like dysplasia in patient #1 demonstrated a *BRAF* p.V600E mutation, whereas a class 3 *BRAF* mutation (p.G469A) was identified in the serrated dysplasia, NOS in patient #8. Both the SSL-like dysplasia (patients #1, #3, and #5) and serrated dysplasia, NOS (patients #4 and #8) showed likely pathogenic variants in *KMT2C* or *EXT1*. However, mutations in *TP53* or *POLG* were found only in the serrated dysplasia, NOS (patients #8 and #11, respectively). In line with the *TP53* mutation in patient #8, immunohistochemistry for p53 showed overexpression (Figure 2B). Patient #6, who had both SSL-like dysplasia and mixed SSL-like/TSA-like dysplasia, exhibited a pathogenic mutation in *MUTYH* (p.R217H), along with mutations in *MADD*.

## 3. Discussion

The clinicopathologic and molecular features of SSL-like dysplasia in IBD are not yet fully understood. In this regard, we note that SSL-like dysplasia was more frequently found in the left colon (60%) and in men (60%) (Table 2), in contrast to its sporadic counterpart, which is typically located in the right colon and more commonly affects women [15,16,17,18,19,20]. Also, the mean age of patients with SSL-like dysplasia (56 years) was lower than the reported age range for sporadic SSL with dysplasia (60–76 years) [15,16,17,18,19,20]. Furthermore, in addition to the typically observed *BRA*F mutation, the *MLH1* mutation was detected in a single case, which may rarely be detected in sporadic SSL with dysplasia [5,17,21]. WES-identified mutations have not been previously reported in SSL with or without dysplasia, including *POLE, KMT2C,* and *EXT1*. Overall, these findings suggest that SSL-like dysplasia in IBD may represent a distinct entity compared with its sporadic counterpart.

*POLE* plays a crucial role in the DNA proofreading mechanism, but its mutations are rare in CRC, occurring in approximately 3% of cases [22,23]. These mutations are more commonly found in younger individuals, affect men more frequently than women, and are usually located in the right colon [22,23,24]. Notably, among Asian patients, 68% of *POLE* mutations are identified in the left colon, whereas 64% of non-Asian patients have them in the right colon (*p* < 0.01) [23]. *POLE* mutations are usually independent of *BRAF* and *KRAS* mutations, as well as MSI [22,23]. The presence of *POLE* mutations in SSL-like dysplasia that lacks *BRAF, KRAS*, or *MLH*1 mutations (patient #3; Table 3) suggests that a subset of SSL-like dysplastic lesions may serve as precursors for *POLE*-mutant CRCs. Although *POLE*-mutant CRCs are generally considered a subtype with a favorable prognosis, they can exhibit high-grade histologic features, including poorly cohesive rhabdoid cells and areas resembling medullary carcinoma [22,23,25]. Similarly, mutations in genes involved in histone modification, such as *KMT2C*, are exceedingly rare in CRC, occurring in approximately 1% of cases, but are significantly more frequent in CRC affecting younger patients [26]. Meanwhile, *EXT1* is primarily recognized for its role in hereditary multiple exostoses (osteochondromas), and the potential relevance of *EXT1* mutations in CRC remains unclear [27]. Of note, although rare, mutations in *KRAS* and *PTEN* have been observed in sporadic SSL with dysplasia. Bettington et al. identified *KRAS* mutations at codon 12 or 13 in 1 (1%) out of 137 SSLs with dysplasia, while Murakami et al. reported PTEN mutations in 1 (13%) out of 8 SSLs with dysplasia [17,21]. Consistent with these findings, only one (25%) of the four SSL-like dysplastic lesions in our cohort had both mutations (patient #5) (Table 3). However, this lesion also exhibited a likely pathogenic variant in *MLH1*, along with a high number of pathogenic and likely pathogenic variants, most of which were small frameshift insertions or deletions, indicating MSI. Indeed, a previous study showed that mutations in various genes, including *PTEN*, could result from MSI in MSI-high CRCs [28].

Serrated dysplasia, NOS is a recently described subtype of serrated dysplasia that lacks the typical features of SSL-like dysplasia or TSA-like dysplasia. However, its clinicopathologic and molecular features are largely unknown. In this regard, we found that the serrated dysplasia, NOS predominantly occurred in the left colon (83%) and was more common in men (80%) (Table 2). Notably, the serrated dysplasia, NOS exhibited a higher frequency of HGD (50%) compared with the SSL-like dysplasia (20%). The serrated dysplasia, NOS was also more frequently associated with nonconventional dysplasia (60%, including 2 cases of hypermucinous dysplasia and a single case of goblet cell-deficient dysplasia) compared with the SSL-like dysplasia (20%, including one case of mixed SSL-like/TSA-like dysplasia). Nonconventional dysplasia, including hypermucinous dysplasia, crypt dysplasia (which features mild cytologic atypia confined to the crypt bases), and goblet cell-deficient dysplasia, is considered high risk because they are more frequently detected as flat/invisible dysplasia (42–100%) and associated with advanced neoplasia upon follow-up (40–93%) compared with conventional dysplasia or sporadic adenomas (18% and 10%, respectively) [2,9,29,30,31,32,33,34]. Consistently, the serrated dysplasia, NOS demonstrated worse outcomes upon follow-up, with 3 of the 5 patients (60%) developing CRC.

In addition to the mutations observed in the SSL-like dysplasia, such as *BRAF*, *KMT2C*, and *EXT1*, the serrated dysplasia, NOS exhibited mutations in *TP53* or *POLG*. Notably, a single case of serrated dysplasia, NOS (patient #8) harbored both *TP53* and *BRAF* mutations without *MLH1* mutations (Table 3), aligning with previous findings stating that *BRAF*-mutated microsatellite stable (MSS) CRCs frequently present with *TP53* mutations (41% vs. 17% in *BRAF*-mutated, MSI CRCs) and are considered the most aggressive molecular subtype [17,35,36,37]. The significance of *POLG* mutations in another case of serrated dysplasia, NOS (patient #11), which are associated with a wide variety of mitochondrial diseases, remains uncertain, as they have not been previously linked to any serrated lesions. Linkowska et al. also found no evidence supporting a role for *POLG* mutations in driving the accumulation of somatic mutations in mitochondrial DNA or in the development and progression of CRC [38]. However, more recently, using a mouse model of inflammation-induced colon tumorigenesis, Maiuri et al. reported that inflammation-induced alterations in Polg expression may play a significant role in tumorigenesis by reducing mitochondria levels and altering metabolism, making the tumor more resistant to oxidative stress [39]. Taken together, these findings suggest that serrated dysplasia NOS may have distinct molecular features compared with SSL-like dysplasia, and a subset of these lesions could represent precursor lesions for *BRAF*-mutated MSS CRCs, potentially explaining their higher malignant potential.

A potential limitation of our study was that due to the very high number of the original cohort, solely patients with neoplasia diagnosis were reevaluated; therefore, samples may have been missed during the reevaluation process. Our findings support the fact that IBD-associated serrated lesions are often associated with CRC (45%), and therefore, may be later on defined in the serrated dysplasia category. Such lesions should be excised completely, and patients may benefit from a closer follow-up [4,40,41].

## 4. Materials and Methods

**Patient data collection**—We retrospectively reviewed data from 2396 patients treated for ulcerative colitis (UC) (n = 1400), Crohn’s disease (CD) (n = 970), and indeterminate colitis (n = 26) at the University of Szeged between 2011 and 2023. Among them, 177 (7%) patients were diagnosed with colorectal neoplasia, of which only 11 (6%) had serrated dysplasia (n = 13) (Figure 1). All serrated dysplastic lesions were reevaluated and subtyped regardless of the sample type (biopsy/resection) by 2 gastrointestinal pathologists (W.T.C. and A.S.) based on the published morphologic criteria [1,2,3,4,10,11,12,13]. For each patient, relevant clinicopathologic information was also collected by reviewing their medical charts and pathology reports. This included age, gender, IBD characteristics (such as subtype, extent, and duration), presence of primary sclerosing cholangitis (PSC), and features of dysplasia (including location, endoscopic appearance, size, and histologic grade). Additionally, data on any concurrent or subsequent dysplasia and colorectal cancer (CRC) were collected. Progression-free survival was defined either as dysplasia recurrence or carcinoma development in the same colon segment, while overall survival was calculated until the end of follow-up or death. This study was approved by the Medical Research Council (BM/28834-1/2024) and the Regional and Institutional Human Biomedical Research Ethics Committee of the University of Szeged (5670).

**WES**—Ten serial sections, each 10 μm thick, were cut from the paraffin block for each sample. Deparaffinization and Proteinase K treatment were performed overnight at 55 °C. The extracted DNA was purified using the 0.5 vol KAPA Pure Bead (Roche, Mannheim, Germany) and eluted in 20 μL of 10 mM Tris-HCl pH 8 buffer. The DNA concentration was determined using the Quant-iT 1x dsDNA HS Assay kit (Thermo Fisher Scientific, Waltham, MA, USA) and measured with a Fluostar Omega (BMG Labtech, Ortenberg, Germany; Software version: 1.01) plate reader. For the library construction, the Twist Library Preparation EF Kit 2.0 with the Universal Adaptor System and Exome 2.0 Panel (Twist Bioscience, South San Francisco, CA, USA) was used, following the manufacturer’s protocol. The fragment size distribution of the pre-capture and post-capture libraries was determined using capillary electrophoresis on the LabChip GX Touch HT Nucleic Acid Analyzer, employing the X-Mark HT Chip and the DNA NGS 3K Assay kit (PerkinElmer, Waltham, MA, USA). The libraries were quantified using the Quant-iT 1x dsDNA HS Assay kit (Thermo Fisher Scientific, Waltham, MA, USA) with Fluostar Omega (BMG Labtech, Ortenberg, Germany). The pooled libraries were diluted to 1.5 nM and prepared for 2 × 150 bp paired-end sequencing using the 300-cycle S4 Reagent Kit on the NovaSeq 6000 Sequencing System (Illumina, San Diego, CA, USA), following the manufacturer’s protocol. On average, more than 24 Gbp of raw data was generated per sample. Demultiplexing, adapter trimming, Q30-filtering, and somatic variant calling of the sequenced data were performed on the Dragen Bio-IT platform (Illumina, San Diego, CA, USA). Genomic variants of Vcf files were annotated using the Nirvana Software package (OncoKB Database) [42]. Variants were manually reviewed for the read quality and the number of reads. The variants had to show at least 10 variant reads to be considered for further analysis. Pathogenic and likely pathogenic reads in genes with known oncogenic or tumor suppressor activity were included in the results (ClinVar Database) [43]. Also, variants that resulted in a frameshift, non-sense (stop-gain), splice donor/splice acceptor site altering, or transcription start site altering in the tumor suppressor genes (OncoKB Database) were included [42].

## 5. Conclusions

In conclusion, serrated dysplasia was rare in IBD, with a prevalence of 6%. While morphologically identical to its sporadic counterpart, SSL-like dysplasia in IBD could represent a distinct entity with different clinical and molecular features. Additionally, serrated dysplasia, NOS exhibited different molecular profiles compared with SSL-like dysplasia and may have a higher malignant potential. Further studies involving larger cohorts are needed to validate our findings.

## Figures and Tables

**Table 1:** Clinicopathologic features of the IBD patients with serrated dysplasia.

	Serrated dysplasia (n = 13, from 11 patients)
**Mean age (years, range)**	56 (35–71)
**Male gender (%)**	8 (73%)
**IBD subtype (%)**	10 UC (91%) 1 CD (9%)
**Extent of IBD (%)**	7 pancolitis (64%) 4 left-sided (36%)
**Mean duration of IBD (years, range)**	26 (5–59)
**Primary sclerosing cholangitis (%)**	0 (0%)
**Subtype of serrated dysplasia (%)**	5 SSL-like dysplasia (38%) 1 TSA-like dysplasia (8%) 6 serrated dysplasia, NOS (46%) 1 mixed SSL-like/TSA-like dysplasia (8%)
**Location of dysplasia (%)**	9 left (69%) 4 right (31%)
**Endoscopic appearance of dysplasia** (%)	11 polypoid (85%) 2 flat (15%)
**Mean size of dysplasia (cm, range)**	0.8 (0.2–2.5)
**Histologic grade of dysplasia (%)**	8 LGD (62%) 5 HGD (38%)
**Association with conventional dysplasia (%)**	3 patients (27%)
**Association with nonconventional dysplasia (%)**	3 patients (27%)
**Association with CRC (%)**	5 patients (45%)

**Abbreviations:** CD, Crohn’s disease; CRC, colorectal cancer; HGD, high-grade dysplasia; IBD, inflammatory bowel disease; LGD, low-grade dysplasia; NOS, not otherwise specified; SSL, sessile serrated lesion; TSA, traditional serrated adenoma; UC, ulcerative colitis.

**Table 2:** Clinicopathologic features of the serrated dysplastic subtypes.

	SSL-like Dysplasia (n = 5, from 5 Patients)	Serrated Dysplasia NOS (n = 6, from 5 Patients)	TSA-like Dysplasia (n = 1, from 1 Patient)	Mixed SSL-like/TSA-like Dysplasia (n = 1, from 1 Patient)
**Mean age (years, range)**	56 (35–71)	52 (36–63)	53	71
**Male gender (%)**	3 (60%)	4 (80%)	1 (100%)	1 (100%)
**IBD subtype (%)**	5 UC (100%) 0 CD (0%)	4 UC (80%) 1 CD (20%)	1 UC (100%) 0 CD (0%)	1 UC (100%) 0 CD (0%)
**Extent of IBD (%)**	4 Pancolitis (80%) 1 Left-sided (20%)	3 Pancolitis (60%) 2 Left-sided (40%)	0 Pancolitis (0%) 1 Left-sided (100%)	1 Pancolitis (100%) 0 Left-sided (0%)
**Mean duration of IBD** **(years, range)**	31 (5–59)	30 (7–47)	44	8
**Location of dysplasia (%)**	3 Left (60%) 2 Right (40%)	5 Left (83%) 1 Right (17%)	1 Left (100%) 0 Right (0%)	0 Left (0%) 1 Right (100%)
**Endoscopic appearance of dysplasia** (%)	5 Polypoid (100%) 0 Flat (0%)	5 Polypoid (83%) 1 Flat (17%)	1 Polypoid (100%) 0 Flat (0%)	0 Polypoid (0%) 1 Flat (100%)
**Mean size of dysplasia** **(cm, range)**	0.5 (0.2–0.9)	1.0 (0.3–2.5)	0.6	0.5
**Histologic grade of dysplasia (%)**	4 LGD (80%) 1 HGD (20%)	3 LGD (50%) 3 HGD (50%)	0 LGD (0%) 1 HGD (100%)	1 LGD (100%) 0 HGD (0%)
**Association with conventional dysplasia (%)**	1 Patient with TA-like dysplasia (20%)	1 Patient with TVA-like dysplasia (20%)	0 Patient (0%)	1 Patient with TA-like dysplasia (100%)
**Association with nonconventional dysplasia (%)**	1 Patient with mixed SSL-like/TSA-like dysplasia (20%)	2 Patients with hypermucinous dysplasia (40%) 1 Patient with goblet cell-deficient dysplasia (20%)	0 Patient (0%)	1 Patient with SSL-like dysplasia (100%)
**Association with CRC (%)**	1 Patient (20%)	3 Patients (60%)	1 Patient (100%)	0 Patient (0%)

**Table 3:** Molecular features of serrated dysplasia.

Patient #	Subtype	Pathogenic Mutations	Likely Pathogenic Mutations
1	SSL-like dysplasia	*BRAF, ATR*	*KMT2C*
3	SSL-like dysplasia	*EXT1*	*POLE, CDKN1B*
4	Serrated dysplasia NOS	*-*	*KMT2C, MAX, CDC6*
5	SSL-like dysplasia	*KRAS, PTEN, TSC1*	*MLH1, DICER1, HIF1A, ASXL1, ZNF292, EXT1, ACVR2A, KMT2C, MGA, SETD1B, TGFBR2*
6 (lesion #1)	SSL-like dysplasia	*MUTYH, MADD*	*SERPINB4*
6 (lesion #2)	Mixed SSL-like/TSA-like dysplasia	*MUTYH, MADD*	*-*
8	Serrated dysplasia NOS	*-*	*TP53, BRAF, MPL, EXT1*
11	Serrated dysplasia NOS	*POLG*	-

Abbreviations: NOS, not otherwise specified; SSL, sessile serrated lesion; TSA, traditional serrated adenoma.

## Data Availability

The data that support the findings of this study are available upon request from the corresponding author, Anita Sejben.

## Abbreviations

The following abbreviations are used in this manuscript:

CD	Crohn’s disease
CRC	Colorectal carcinoma
IBD	Inflammatory bowel disease
NOS	Not otherwise specified
PSC	Primary sclerosing cholangitis
SSL	Sessile serrated lesion
TSA	Traditional serrated adenoma
UC	Ulcerative colitis
WES	Whole-exome sequencing
